# Gestational diabetes mellitus in Cameroon: prevalence, risk factors and screening strategies

**DOI:** 10.3389/fcdhc.2023.1272333

**Published:** 2024-01-09

**Authors:** Eugene Sobngwi, Joelle Sobngwi-Tambekou, Jean Claude Katte, Justin B. Echouffo-Tcheugui, Eric V. Balti, Andre-Pascal Kengne, Leopold Fezeu, Chobufo Muchi Ditah, Alain-Patrick Tchatchoua, Mesmin Dehayem, Nigel C. Unwin, Judith Rankin, Jean Claude Mbanya, Ruth Bell

**Affiliations:** ^1^ Department of Internal Medicine and Specialities, Faculty of Medicine and Biomedical Sciences, University of Yaoundé I, Yaoundé, Cameroon; ^2^ Laboratory of Molecular Medicine and Metabolism, Biotechnology Center, University of Yaoundé I, Yaoundé, Cameroon; ^3^ Department of Non-Communicable Diseases, Recherche Santé et Développement (RSD) Institute, Yaoundé, Cameroon; ^4^ Department of Clinical and Biomedical Sciences, University of Exeter Medical School, Exeter, United Kingdom; ^5^ Hubert Department of Global Health, Rollins School of Public Health, Emory University, Atlanta, GA, United States; ^6^ Division of Endocrinology, Diabetes and Hypertension, Brigham and Women’s Hospital, Harvard Medical School, Boston, MA, United States; ^7^ Diabetes Research Center, Faculty of Medicine and Pharmacy, Brussels Free University-Vrije Universiteit Brussel (VUB), Brussels, Belgium; ^8^ Non-Communicable Diseases Research Unit, South African Medical Research Council and University of Cape Town, Cape Town, South Africa; ^9^ Nutritional Epidemiology Research Unit-UMR U557 Institut National de la Santé et de la Recherche Médicale (INSERM), U1125 INRA, CNAM, University of Paris 13, Bobigny, France; ^10^ Faculty of Medical Sciences, Public Health and Epidemiology, University of the West Indies at Cave Hill, Bridgetown, Barbados; ^11^ Institute of Health & Society, Newcastle University, Newcastle upon Tyne, United Kingdom

**Keywords:** gestational diabetes mellitus, screening, prevalence, risk factors, Cameroon, sub-Saharan Africa

## Abstract

**Background:**

The burden of gestational diabetes (GDM) and the optimal screening strategies in African populations are yet to be determined. We assessed the prevalence of GDM and the performance of various screening tests in a Cameroonian population.

**Methods:**

We carried out a cross-sectional study involving the screening of 983 women at 24-28 weeks of pregnancy for GDM using serial tests, including fasting plasma (FPG), random blood glucose (RBG), a 1-hour 50g glucose challenge test (GCT), and standard 2-hour oral glucose tolerance test (OGTT). GDM was defined using the World Health Organization (WHO 1999), International Association of Diabetes and Pregnancy Special Group (IADPSG 2010), and National Institute for Health Care Excellence (NICE 2015) criteria. GDM correlates were assessed using logistic regressions, and *c*-statistics were used to assess the performance of screening strategies.

**Findings:**

GDM prevalence was 5·9%, 17·7%, and 11·0% using WHO, IADPSG, and NICE criteria, respectively. Previous stillbirth [odds ratio: 3·14, 95%CI: 1·27-7·76)] was the main correlate of GDM. The optimal cut-points to diagnose WHO-defined GDM were 5·9 mmol/L for RPG (*c*-statistic 0·62) and 7·1 mmol/L for 1-hour 50g GCT (*c*-statistic 0·76). The same cut-off value for RPG was applicable for IADPSG-diagnosed GDM while the threshold was 6·5 mmol/L (*c*-statistic 0·61) for NICE-diagnosed GDM. The optimal cut-off of 1-hour 50g GCT was similar for IADPSG and NICE-diagnosed GDM. WHO-defined GDM was always confirmed by another diagnosis strategy while IADPSG and GCT independently identified at least 66·9 and 41·0% of the cases.

**Interpretation:**

GDM is common among Cameroonian women. Effective detection of GDM in under-resourced settings may require simpler algorithms including the initial use of FPG, which could substantially increase screening yield.

## Introduction

Despite progress in maternal and child health globally, adverse pregnancy outcomes remain unacceptably high in most sub-Saharan African countries (1), mainly due to preventable causes (2). Gestational diabetes mellitus (GDM), one of those preventable causes, disproportionately affects women of African ethnicity (3). Diabetes mellitus has been associated with up to 8% stillbirths in developed countries against less than 2% in other parts of the world (1, 4). However, the latter figures are likely underestimated, as the true magnitude of GDM remains unknown in most low- to-middle income countries (LMICs), including those in Africa (4). Recent findings show that only about 30% of African women undergo screening for diabetes during pregnancy (5, 6).

Detecting and treating GDM have benefits in reducing fetal morbidity and mortality, as well as the future maternal risk of developing type 2 diabetes (7, 8). However, optimal strategies for GDM diagnosis remain elusive, and no general consensus exists on the subject (9). Different professional bodies have proposed different diagnostic criteria. The International Association of Diabetes and Pregnancy Study Groups (IADPSG) is widely used, informed mainly by the Hyperglyaemia and Adverse Pregnancy Outcome (HAPO) study. However, the HAPO study identified continuous associations between maternal glucose levels and several perinatal outcomes, extending the threshold for GDM diagnosis (10).

In most developed countries, women of African ethnicity are offered universal screening for GDM due to their elevated risk of the disease. Strategies for GDM screening in developed countries may not be suitable for the African setting, a context with competing health priorities. Adequate approaches should account for the health systems’ overall population risk and coping capacity. Awareness of risk factors such as family history of diabetes is as low as 30% in most sub-Saharan African settings (11). Moreover, diagnosing GDM requires the resource- and labor-intensive oral glucose tolerance test (OGTT), which is seldom performed in African settings due to many constrains (12, 13). Consequently, GDM is either not screened at all or is screened using urine glucose, which has been demonstrated to be inaccurate compared to the OGTT (14).

In the current study, we assessed the prevalence and correlates of GDM among pregnant Cameroonian women according to different recommended diagnostic criteria. Additionally, we assessed the performance and yield of various screening tests or strategies for GDM.

## Methods

### Study design and setting

This is a cross-sectional analysis from the GDM Cameroon Study (Improving screening and management of gestational diabetes in Cameroon), which enrolled consenting pregnant women aged above 18 years who attended antenatal care at the participating health facilities: Central Maternity of the Yaoundé Central Hospital and the Antenatal Clinic of the Bamenda Regional Hospital. The Yaoundé Central Hospital is a Public Tertiary Teaching Hospital,and one of the Capital City’s referral centers, with an average of 300 monthly deliveries (15). The Bamenda Regional Hospital is a public secondary care and regional referral facility covering the North-Western region of Cameroon. The two facilities were selected to represent the diversity of women in Cameroon, regarding socio-economic levels, urbanity, and potential GDM severity. The study was approved by the National Ethics Committee of Cameroon, and all participants gave written informed consent. The present report complied with the strengthening of the reporting of observational studies in epidemiology (STROBE) statement.

### Participants and study procedures

The GDM Cameroon study enrolled consenting pregnant women (24 – 28 weeks of gestation) between January and June 2009, who attended routine antenatal care at the participating health facilities, on a consecutive basis until reaching a minimum screening target sample of 1000. We excluded women with known diabetes based on medical records. Overall, each consenting participant underwent a risk factors assessment, random blood glucose (RBG), fasting plasma glucose (FPG), 1-hour 50 g glucose challenge test (GCT), and 2-hour 75g OGTT.

#### Risk factors assessment

After obtaining informed consent, a structured questionnaire was used to record risk factors for GDM, including age, occupation, education level, parity, history of previous stillbirth, history of macrosomia (birth weight ≥4,000 g), physical activity levels, dietary habits, and characteristics of the ongoing pregnancy. Blood pressure was the average from three consecutive measurements in a sitting position after a 10-minute rest using an Omron M4^®^ recorder. Height and weight were measured in light indoor clothing and without shoes. The body mass index (BMI) was calculated in kg/m^2^. Overweight was defined as BMI ≥25kg/m² and obesity as BMI ≥30kg/m².

#### Testing for gestational diabetes

The women underwent biochemical testing for GDM on two different occasions. Random plasma glucose (RPG) and 1-hour post load 50g glucose challenge test (GCT) were conducted at the first visit. At the second visit, within one week of the first, participants underwent testing (after an 8-12 hour overnight fast) including FPG and a 75g OGTT with assessment of blood glucose at 30 and 120 minutes after glucose load.

#### Biochemical measurements

Plasma glucose levels were measured using the point-of-care HemoCue B-glucose analyzer based on the glucose dehydrogenase reaction (HemoCue AB, Angelholm, Sweden). The stability of the analyses was checked daily, and external calibration with the quality assurance scheme was undertaken monthly. We did not assess the performance of the HemoCue B-glucose analyzer in this study although other previous studies have reported a high accuracy and robustness with plasma glucose measured using standard laboratory-based glucose oxidase methodologies (16, 17).

#### Diagnostic criteria for GDM

Gestational diabetes was defined by applying three sets of criteria: 1) the World Health Organization (WHO) definition 1999 as either FPG ≥ 7·0 mmol/L or 2-hour post 75g OGTT plasma glucose (2h PG) ≥ 7·8 mmol/L (14); 2) an approximation of the IADPSG criteria as either FPG ≥ 5·1 mmol/L or 2h PG ≥ 8·5 mmol/L (15); and 3) the NICE 2015 criteria as FPG ≥ 5·6 mmol/L and 2h PG≥ 7·8 mmol/L (16).

### Statistical methods

Continuous variables were summarized as mean and standard deviation (SD), and categorical variables as counts and percentages. The various estimates of GDM prevalence were based on the WHO, IADPSG, or NICE criteria. Logistic regression models were used to investigate correlates of GDM. The sensitivity, specificity, and predictive values of screening tests at commonly advocated thresholds to diagnose GDM were determined against the three GDM definitions for RBG, FPG, and 1-hour post 50g GCT and blood glucose two hours after oral glucose stimulation. After that, receiver operating characteristic curves analyses were used to derive the optimal threshold for each test to diagnose GDM definition based on the Youden’s J point. The derived cut-point was tested across the WHO, IADPSG, and NICE-diagnosed GDM. The concordance between the various screening strategies was illustrated using Venn diagrams. All analyses were performed using Stata version 14·0 (StataCorp LP, Texas, USA). A p-value of ≤0·05 was used to characterize statistically significant results.

### Screening algorithm

Based on the performance of the individual screening tests and their combination, we derived and proposed a screening algorithm for resource-limited settings using capillary glucose measurement.

### Role of the funding source

The funders had no role in the study design, data collection, analysis, interpretation, or writing of the report. The corresponding author had full access to all the study data and final responsibility for submission for publication.

## Results

### Characteristics of the study population

1,013 women were invited to participate in the study, 983 (97%) attended the screening, and 938 had complete data on all the variables of interest. The main reason for non-participation was the lack of time to attend all the study appointments. **Supplementary Table 1** summarizes the characteristics of participants. The mean age was 25·5 (standard deviation [SD]:5·3) years, and the mean gestational age at inclusion was 25·6 (SD: 1·7) weeks. The majority of women had a primary level of education, were married or living in a couple, exercised less than 30 minutes a week, and were overweight or obese. Of the women included, 42·5% were primigravidae, 38·1% reported having had a blood glucose test done at least once in the previous year, and 30·3% reported never having a blood glucose test before the study (not shown).

### Prevalence of gestational diabetes mellitus

The prevalence of GDM using the WHO 1999, IDASPG 2010, or NICE 2015 criteria is shown in **Table 1**. Based on the WHO 1999 diagnostic criteria, the prevalence of GDM was 5·9%, with a minority of participants having a FPG ≥ 7·0 mmol/L (0·8%). Using the IADPSG diagnostic criteria, the GDM prevalence was 17·7%, with 16·8% diagnosed by FPG (≥ 5·1 mmol/L) only. When NICE 2015 criteria were applied, GDM was identified in 11·0% of the study population, while diagnosis based on FPG represented 7·1%.

**Table 1:** Prevalence of gestational diabetes.

Criteria for definitions of gestational diabetes	WHO 1999		IADPSG 2010		NICE 2015	
	Threshold	Prevalence n (%)	Threshold	Prevalence n (%)	Threshold	Prevalence n (%)
Fasting plasma glucose (FPG)	7·0 mmol/L	7 (0·8)	5·1 mmol/L	159 (16·8)	5·6 mmol/L	66 (7·1)
Two-hour post-glucose load glycaemia (2h-PG)	7·8 mmol/L	49 (5·3)	8·5 mmol/L	17 (1·8)	7·8 mmol/L	49 (5·3)
FPG and/or 2h-PG	–	55 (5·9)	–	165 (17·7)	–	102 (11·0)

### Correlates of gestational diabetes mellitus

**Table 2** shows the prevalence of GDM across strata of putative risk factors and the accompanying odds ratio for the three definitions of GDM. In age-adjusted analyses, IADPSG-defined GDM differed by status for alcohol consumption (p=0·035). At the same time, WHO-defined GDM increased with increasing walking time per week (p=0·001) with evidence of a linear trend (p=0·006, not shown). None of the characteristics was simultaneously associated with WHO- and IADPSG-defined GDM. Being current drinkers relative to non-drinkers of alcohol was associated with a 55% (95%CI 3-132%) higher odds. Medical history of stillbirths was associated with a 214% (95%CI 27 - 676%) increase in GDM defined by NICE 2015 criteria.

**Table 2:** Prevalence of gestational diabetes by categories of putative risk factors after adjustment for age.

Characteristics	%	WHO 1999	%	IADPSG 2010	%	NICE 2015
		OR (95% CI)		OR (95% CI)		OR (95% CI)
Alcohol consumption						
Non drinker	38·2	1·00	42·2	1·00	41·2	1·00
Ex-drinkers	25·5	1·60 (0·84 – 3·06)	23·5	0·84 (0·54 – 1·29)	25·5	0·93 (0·55 – 1·57)
Current drinkers	36·4	1·02 (0·51 – 2·05)	34·3	1·55 (1·03 – 2·32) ^§^	33·3	1·41 (0·86 – 2·31)
Family history DM						
No	83·7	1·00	84·9	1·00	82·6	1·00
Yes	16·3	1·03 (0·47 – 2·27)	15·1	0·93 (0·56 – 1·53)	17·4	1·15 (0·64 – 2·05)
History of macrosomia						
No	83·6	1·00	89·8	1·00	89·2	1·00
Yes	16·4	1·77 (0·83 – 3·79)	10·2	0·99 (0·56 – 1·76)	10·8	1·08 (0·55 –2·12)
Number of live births						
0	38·9	1·00	40·6	1·00	41·6	1·00
1 – 2	35·2	0·90 (0·46 – 1·78)	43·6	1·10 (0·74 – 1·64)	36·6	0·87 (0·53 – 1·44)
≥ 3	25·9	2·26 (0·90 – 5·68)	15·8	1·29 (0·69 – 2·40)	21·8	1·67 (0·82 – 3·42)
History of stillbirths						
No	92·7	1·00	96·4	1·00	93·1	1·00
Yes	7·3	2·93 (0·96 – 8·95)	3·6	1·47 (0·57 – 3·76)	6·9	3·14 (1·27 – 7·76) ^†^
Walking per week						
< 30 min	36·4	1·00	53·1	1·00	47·1	1·00
30 – 60 min	32·7	1·70 (0·88 – 3·28)	28·8	0·98 (0·65 – 1·46)	30·4	1·13 (0·69 – 1·83)
> 60 min	30·9	3·05 (1·55 – 6·00) ^‡^	18·1	1·19 (0·74 – 1·91)	22·5	1·69 (0·99 – 1·83)^**^
Gestational BMI categories						
Normal weight	15·1	1·00	22·8	1·00	18·0	1·00
Overweight	56·6	1·72 (0·77 – 3·85)	48·8	0·98 (0·63 – 1·52)	57·0	1·42 (0·81 – 2·50)
Obese	28·3	1·92 (0·77 – 4·78)	28·4	1·40 (0·84 – 2·33)	25·0	1·44 (0·74 – 2·80)
High blood pressure						
No	98·1	1·00	99·4	1·00	99·0	1·00
Yes	1·9	1·28 (0·16 – 1·03)	0•6	0·34 (0·05 – 2·83)	1·0	0·65 (0·08 – 5·06)

^†^P=0·006; ^‡^ P=0·001: ^§^ P=0·035; ** P=0·054.

WHO, World Health Organization; IADPSG, International Association of Diabetes and pregnancy study groups; NICE, National Institute for health Care Excellence; OR, odds ratio; CI, confidence interval; DM, Diabetes mellitus; BMI, body mass index.

### Performance of biochemical screening tests

**Table 3** shows the sensitivity, specificity, likelihood ratios (LR), and *c*-statistics of screening tests to detect GDM based on the WHO 1999, IDASPG 2010, and NICE 2015 definitions. For the 1-hour GCT, the optimal cut-point to diagnose WHO-defined GDM was 7·1 mmol/L, corresponding to a sensitivity of 69·1%, specificity of 71·3%, LR+ of 2·41%, LR- of 0·43%; and *c*-statistic of 0·76 using the WHO criteria. The best performances for IADPSG-defined GDM were obtained at the cut-off value of 5·9 mmol/L: sensitivity 47·6%, specificity 72·5%, LR+ 1·73%, LR- 0·73%; *c*-statistic 0·61. The same cut-off value was obtained for NICE-defined GDM with a 63·7% sensitivity, a 66·8% specificity, a 1·90% LR+, a 0·55% LR- and a *c*-statistic of 0·69 (**Table 3**; **Supplementary Figure 1**).

**Table 3:** Screening performance of glucose at fasting, random and 1h post 50g glucose challenge test in detecting pregnant women with GDM according to three definitions.

GDM diagnostic criteria	Accuracy (%)	Sensitivity (%)	Specificity (%)	LR + (%)	LR - (%)	*c*-statistics (95%CI)
WHO 1999						
1hour post 50g glucose load						0·76 (0·69 – 0·82)
≥ 7·2 mmol/L	74·1	60·0	75·0	2·40	0·53	
≥ 7·8 mmol/L	84·2	41·8	86·9	3·18	0·67	
Optimal cut point (7·1 mmol/L)	71·2	69·1	71·3	2·41	0·43	
Fasting plasma glucose						0·74 (0·67 – 0·81)
≥ 5·6 mmol/L	91·2	34·6	94·7	6·49	0·69	
≥ 5·1 mmol/L	82·9	47·3	95·2	3·19	0·62	
Optimal cut point (4·9 mmol/L)	79·1	55·6	80·6	2·82	0·56	
Random blood glucose						0·62 (0·54 – 0·71)
≥ 7·8 mmol/L	93·2	10·9	98·3	6·40	0·91	
≥ 8·0 mmol/L	93·6	9·1	98·1	8·00	0·92	
Optimal cut point (5·9 mmol/L)	74·1	45·4	75·9	1·90	0·72	
IADPSG 2010						
1hour post 50g glucose load						0·61 (0·56 – 0·66)
≥ 7·2 mmol/L	69·5	40·4	75·8	1·67	0·79	
≥ 7·8 mmol/L	75·6	22·9	86·9	1·75	0·89	
Optimal cut point (6·9 mmol/L)	68·1	47·6	72·5	1·73	0·73	
Fasting plasma glucose						0·98 (0·96 – 0·99)
≥ 7·0 mmol/L	83·2	4·8	100·0	na	0·95	
≥ 5·6 mmol/L	89·3	39·8	100·0	na	0·60	
Optimal cut point (5·1 mmol/L)	99·0	94·6	100·0	na	0·05	
Random blood glucose						0·58 (0·53 – 0·63)
≥ 7·8 mmol/L	82·8	7·8	99·0	7·56	0·93	
≥ 8·0 mmol/L	82·6	5·4	99·2	6·98	0·95	
Optimal cut point (5·9 mmol/L)	70·2	37·4	77·2	1·64	0·81	
NICE 2015						
1hour post 50g glucose load						0·69 (0·63 – 0·74)
≥ 7·2 mmol/L	73·4	52·0	76·0	2·16	0·63	
≥ 7·8 mmol/L	81·3	32·4	87·3	2·55	0·77	
Optimal cut point (6·9 mmol/L)	66·2	63·7	66·8	1·90	0·55	
Fasting plasma glucose						0·88 (0·83 – 0·92)
≥ 7·0 mmol/L	90·0	7·8	100·0	na	0·92	
≥ 5·1 mmol/L	88·0	71·6	90·0	7·12	0·32	
Optimal cut point (5·6 mmol/L)	95·2	65·7	99·2	78·45	0·35	
Random blood glucose						0·61 (0·54 – 0·67)
≥ 7·8 mmol/L	88·8	8·8	98·6	6·15	0·93	
≥ 8·0 mmol/L	88·8	5·9	98·9	5·46	0·95	
Optimal cut point (6·5 mmol/L)	83·4	28·4	90·1	2·86	0·89	

LR+: positive likelihood ratio; LR-: negative likelihood ratio; AUC, area under the curve; CI, confidence interval; WHO, World Health Organization; IADPSG, International Association of Diabetes and pregnancy study groups; NICE, National Institute for health Care Excellence; na, not applicable.

**Figure 1:**
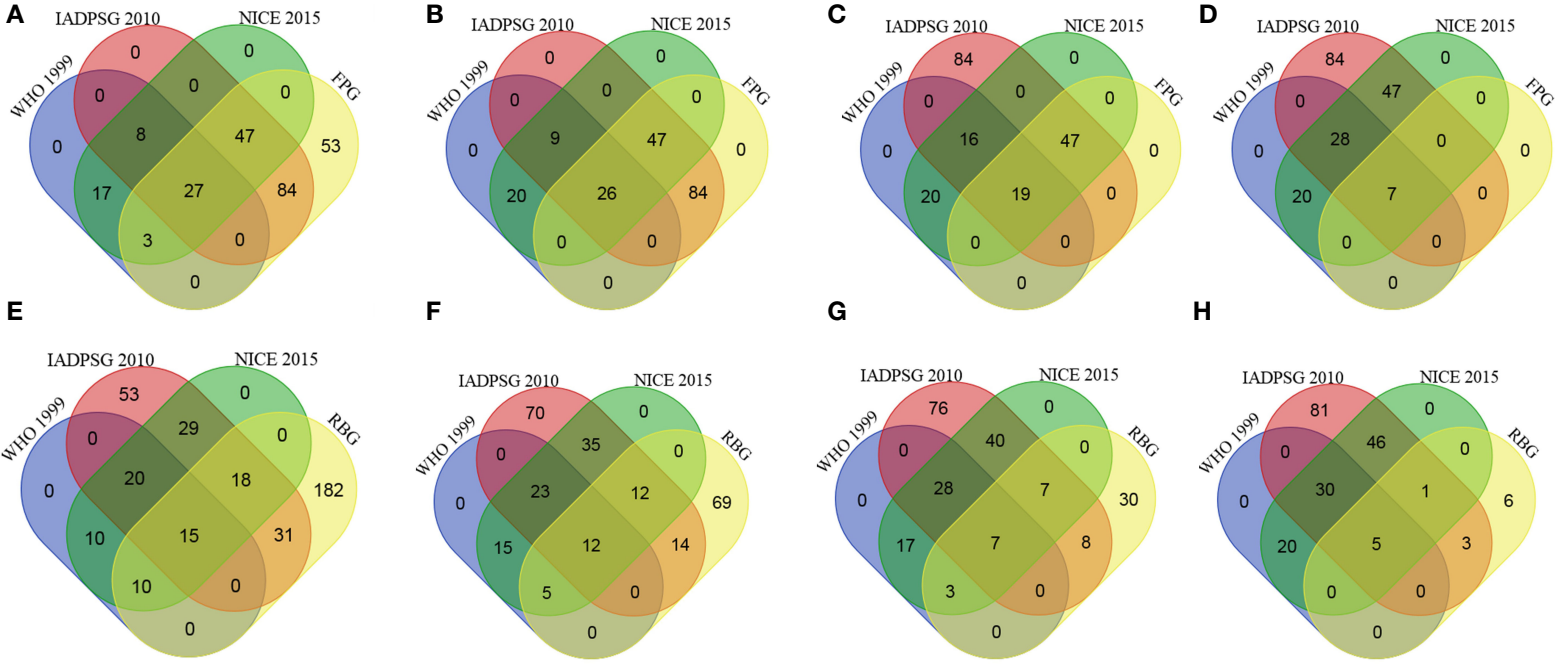
Representation of the number of women identified using WHO 1999, IADPSG 2010 and NICE 2015 criteria compared to fasting plasma glucose (FPG, upper panel) using cut-off values of 4·9 mmol/L **(A)**, 5·1 mmol/L **(B)**, 5·6 mmol/L **(C)** and 7·0 mmol/L **(D)** and compared to random blood glucose value (RBG, lower panel) considering cut-off values of 5·9 mmol/L **(E)**, 6·5 mmol/L **(F)**, 7·8 mmol/L **(G)** and 8·0 mmol/L **(H)**.

The optimal cut-off values for FPG were 5·9, 5·1, and 5·6 mmol/L for WHO, IADPSG and NICE criteria. At these thresholds, FPG achieved a minimal accuracy of 79·1% while the associated sensitivities and specificities were 55·6 and 80·6% for WHO (*c*-statistic of 0·74), 94·6 and 100% for IADPSG (*c*-statistic of 0·98), and 94·6 and 100% for NICE (*c*-statistic of 0·88) diagnosed GDM. Compared to the three diagnostic strategies, as opposed to the threshold of 4·9 mmol/L, none of the study participants was identified with GDM using isolated FPG at the cut-off value of or above 5·1 mmol/L. This group represented 95·6% of IADPSG-diagnosed GDM. By using this glucose value for diagnosis of GDM, 29/186 (15·6%) of women identified using NICE and/or WHO criteria were missed (**Figure 1**). Among those, 23/29 (79·3%) women had at least one risk factor other than their black-African origin, including high BMI (n=7), age>25 years (n=13), family history of type 2 diabetes (n=6), past medical history of macrosomia (n=3) or stillbirth (n=2).

For RBG, the optimal cut-point was 5·9 mmol/L for WHO-defined GDM, and corresponding performance measures were 45·4% (sensitivity), 75·9% (specificity), 1·90% (LR+), 0·72% (LR-), and 0·62 (*c*-statistic). Equivalent performance measures for IADPSG-defined GDM at the same cut-point were 37·4% (sensitivity), 77·2% (specificity), 1·64% (LR+), 0·81% (LR-) and 0·58 (*c*-statistic). The optimal cut-off value obtained for NICE-defined GDM was 6·5 mmol/L and the corresponding sensitivity was 28·4%, specificity 90·1%, LR+ 2·86%, LR- 0·89%; and *c*-statistic 0·61 (**Table 3**). Unlike FPG, despite a good specificity of RBG tested in the overall study population at various cut-off values (5·9, 6·5, 7·8 and 8·0 mmol/L) as shown in **Table 3**, its performance among women identified with GDM by at least one of the testing strategies (including RBG) is somewhat mitigated. Indeed, at the lowest cut-off value (5·9 mmol/L), 182/256 (71%) diagnosed women using RBG are not confirmed by another criterion. Despite this proportion dropped at higher cut-off values, the ratio of women conjointly identified by RBG and at least one other diagnosis strategy declined from 74/368 (20%) to 9/192 (5%) in the range of tested cut-off values (**Figure 1**).

### Proposed screening strategy

Based on performance mentioned above of the various screening tests and to optimize detection, we propose a practical screening algorithm for resource-limited settings using fasting glucose measurements as shown in **Supplementary Figure 2**. Such a screening strategy, allowing testing of pregnant women first in a fasting state, will facilitate an opportunistic approach to screening and has the potential of maximizing the yield of screening in an environment where regular clinic attendance can be an issue.

## Discussion

The prevalence of GDM in Cameroon varies substantially across diagnostic criteria, from 5·9% by the WHO 1999 criteria to 17·7% by IADPSG criteria and 11·0% by NICE criteria. The presence of GDM was determined by previous obstetrical history and dietary habits. Irrespective of the diagnostic criteria and test thresholds used, a considerable proportion of women were likely to have severe glucose intolerance. A conceptually simple diagnostic algorithm with optimal screening yield can be derived based on the performance of various tests in this population and is potentially helpful for routine clinical practice in resource-limited settings.

### Comparison with other studies and explanation of results

Despite a substantial variation resulting from the diversity of the criteria used, the magnitude of GDM in this study was somewhat comparable to previous reports in developing countries. A study from Nigeria using WHO 1999 criteria to diagnose GDM reported a prevalence of 8·3% (18), whereas a previous South African study found a much lower prevalence (1·5%) with similar criteria in a rural setting (19). Out of Africa, the prevalence of GDM in the Iranian population was reported to be 6·1%, 12·1%, and 18·8% by the ADA, WHO, and ADIPS criteria, respectively (20). The rates of GDM based on the IADPSG criteria in Asian populations have been found to be higher than ours, with a GDM prevalence of 23·0% in Thailand and 25·1% in Singapore (21). The observation that the new IADPSG criteria result in almost three times higher GDM prevalence is similar to findings in Japan (22), United Arab Emirates (23), and Mexico (24). A relatively small sample size study from a separate region in Cameroon using the IADPSG criteria found the prevalence of GDM to be 20.5%, which is not very different from our findings using the same criteria (25). The differences in GDM prevalence in our findings and in the other studies clearly underscore the differences in the screening approaches.

The prevalence of GDM is known to be higher amongst people of non-White ethnic backgrounds (26). The elevated rates of GDM in Cameroonian women reported in our study and other African studies may be directly related to the growing problem of obesity in women in sub-Saharan Africa (27, 28). In our study, only previous stillbirth and alcohol consumption were associated with GDM. There are several possible explanations for the lack of association between traditionally known risk factors and GDM, which include the relatively low rates of some of these risk factors in our sample or the low awareness of these risk factors in our population. The paradoxical association of increased physical activity from walking with a high prevalence of GDM may be related to chance or the subjective nature of physical activity assessment. A possible explanation to this finding may be rebound increase in glycaemia in women who walked to the clinic on the day of their GDM screening especially since they were required to present while fasting on the day of the OGTT. We did not robustly measure physical exercise as such we could not capture the intensity and day-to-day variation of physical activity. Studies have suggested that rigorous exercise before an OGTT could impede insulin response with a tendency towards a higher glycemic response (29). However, other studies have demonstrated an inverse relationship between either vigorous physical activity, or increased weekly physical activity and GDM (30, 31).

Our estimates of the performance of various screening tests differ from those reported in previous studies (23, 32). The observed difference is at least partially related to population structure with potential differential GDM baseline risk, sample size, tests used, and cut-offs. Indeed, many previous studies did not include RBG in their assessment. RBG is a fast, simple, and relatively inexpensive test. However, its accuracy has been less frequently studied than other screening tests, with indications that its performance as a screening test for GDM may be limited. Current evidence suggests that random glucose measurement may not be sufficiently sensitive for screening GDM as a standalone test (14). However, compared to other simple test measures, RBG has been shown to have better accuracy than glycosuria and HbA1c in diagnosing GDM (33). Despite these limitations, from a public health perspective, including FPG as an initial test in a stepwise screening for GDM using a combination of various tests appears as a promising practical approach for detecting GDM in under-resourced settings, where universal screening is not always possible, and opportunistic screening with RBG would lead having more GDM cases diagnosed.

There is an increase to screen for GDM before the traditional 24-28 weeks window. Women with high GDM risk are recommended to be tested at 16-18 weeks (34). While observational findings from studies showing the potential benefit of early GDM screening and treatment show conflicting results, studies especially in native Africans are needed to further investigate the potential benefits with simple screening approaches.

### Strengths and limitations

The strengths of our study include the large sample size and the extensive exploration of cut-offs using multiple diagnostic criteria. To our knowledge, this is one of the largest studies addressing GDM in sub-Saharan Africa, where the burden of the conditions may be increasing. Other strengths include the robust design of the study with an exploration of the performance of several possible GDM detection tests, thus ensuring that the proposed screening algorithm reliably identifies women with GDM in under-resourced settings. The limitations of our study include the absence of measurement of plasma glucose 1 hour after the 75g oral load. Hence, we may have missed some GDM cases. Enrolment was restricted to only two hospitals in the country; thus, results may not readily apply to the general population or in other LMICs. However, to achieve the representativeness of our sample, we ensured the enrolment of women from various socio-economic backgrounds and settings (rural and urban areas). Another limitation was the assessment of the performance of glucose challenge test, FPG and RBG based on existing criteria for diagnosis of GDM instead of adverse maternal and fetal pregnancy outcomes due to the study’s cross-sectional design. Incomplete per/post-partum and post-natal data was a significant constraint to this research endeavor. Lastly, this study was initially conducted in 2009, therefore the data should be interpreted carefully knowing it may not represent today’s situation. However, the findings in this study should inform future studies on GDM in Cameroon and other African settings.

## Conclusion

The GDM prevalence in Cameroon varied substantially with the diagnostic criteria. An opportunistic approach to screening, including an option to use FPG, could considerably increase the yield of screening for GDM. Such an approach appears feasible and acceptable in an under-resourced context.

## Data availability statement

The raw data supporting the conclusions of this article is available upon reasonable request to the corresponding author, sobngwieugene@yahoo.fr.

## Ethics statement

Ethical approval was obtained from the National Ethics Committee before the start of the study. The study was conducted in accordance with all the local institutional requirements. The participants provided their written informed consent before participating in the study.

## Author contributions

ES: Conceptualization, Data curation, Formal Analysis, Funding acquisition, Investigation, Methodology, Project administration, Resources, Supervision, Validation, Visualization, Writing – original draft, Writing – review & editing. JS: Conceptualization, Data curation, Formal Analysis, Funding acquisition, Investigation, Methodology, Project administration, Writing – review & editing. JK: Conceptualization, Data curation, Formal Analysis, Resources, Software, Validation, Visualization, Writing – review & editing. JE: Data curation, Formal Analysis, Investigation, Methodology, Supervision, Writing – review & editing. EB: Conceptualization, Data curation, Formal Analysis, Investigation, Methodology, Writing – review & editing. AK: Conceptualization, Data curation, Formal Analysis, Investigation, Software, Writing – original draft, Writing – review & editing. LF: Conceptualization, Data curation, Investigation, Writing – review & editing. CD: Conceptualization, Data curation, Methodology, Resources, Visualization, Writing – review & editing. AT: Data curation, Methodology, Supervision, Writing – review & editing. MD: Conceptualization, Methodology, Supervision, Writing – review & editing. NU: Project administration, Resources, Software, Supervision, Validation, Visualization, Writing – review & editing. JR: Resources, Supervision, Validation, Visualization, Writing – review & editing. JM: Conceptualization, Resources, Supervision, Validation, Visualization, Writing – review & editing. RB: Resources, Writing – review & editing, Conceptualization.
