# Supplementary figures and images for: Gestational diabetes mellitus in Cameroon: prevalence, risk factors and screening strategies
Source: Front Clin Diabetes Healthc. 2024 Jan 9;4:1272333. doi: 10.3389/fcdhc.2023.1272333 (PMC10876121; doi:10.3389/fcdhc.2023.1272333)

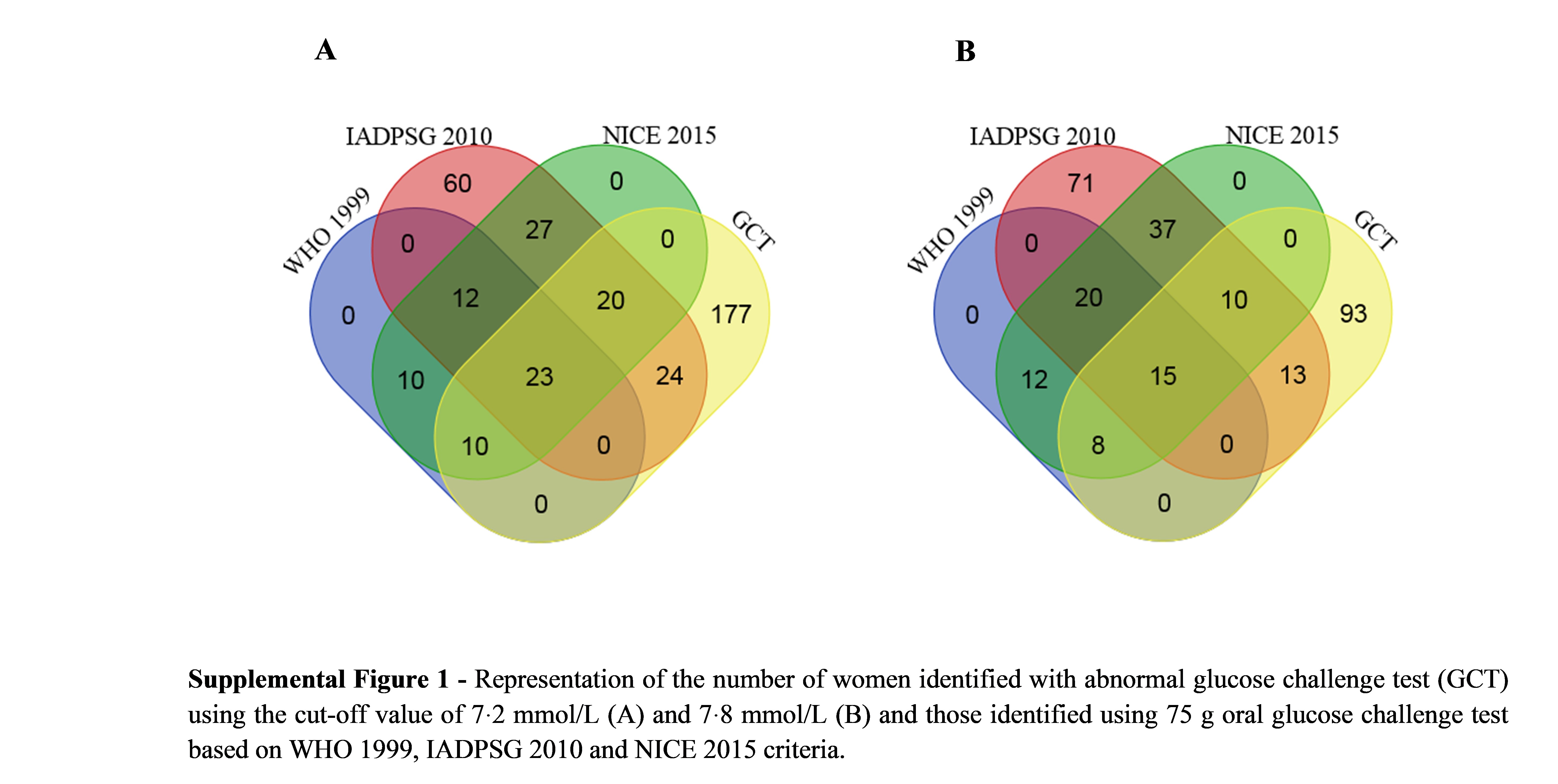

Supplement: Supplementary file 1 [file Image_1.jpeg]

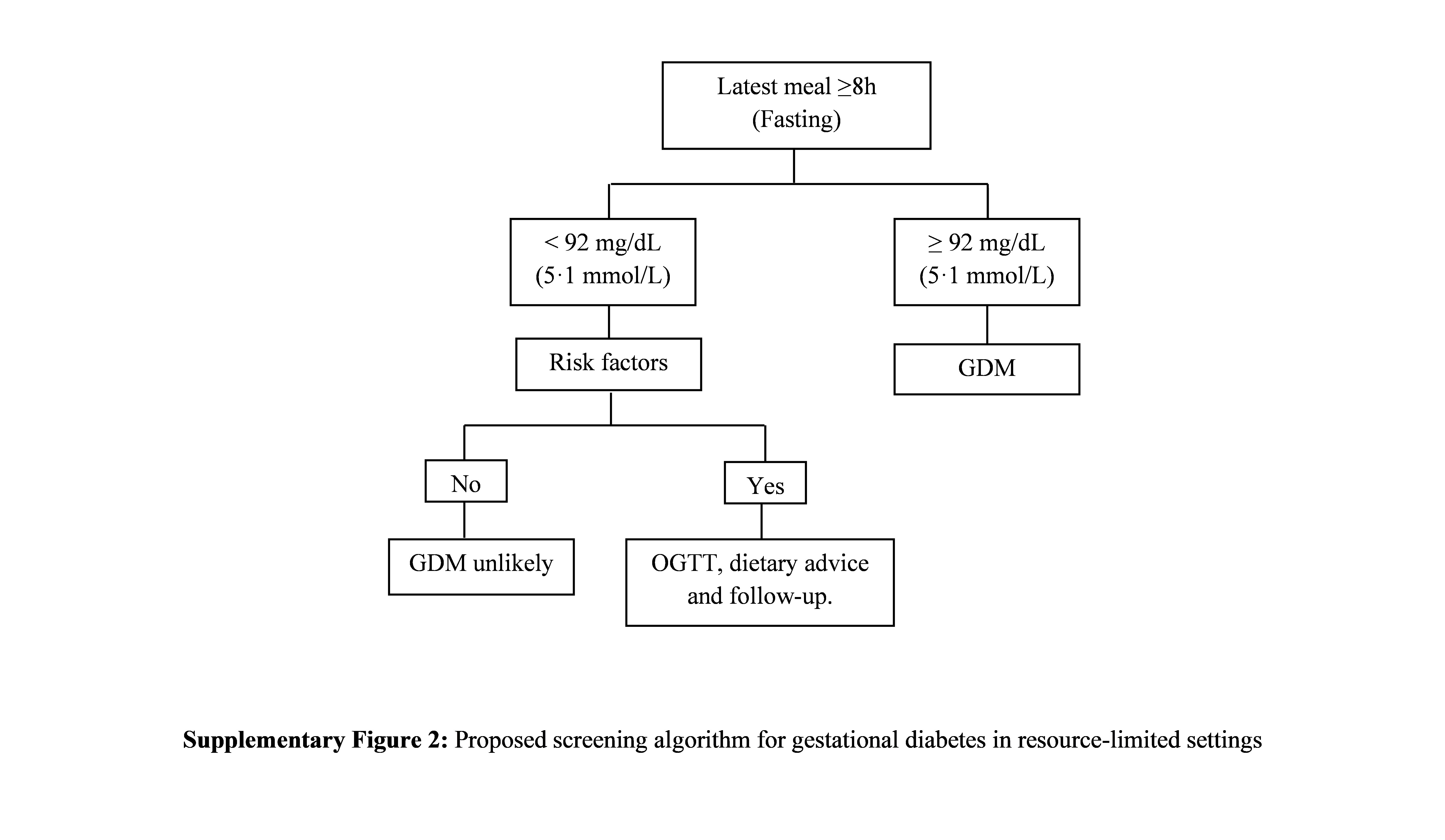

Supplement: Supplementary file 2 [file Image_2.jpeg]
