# Supplementary material for: Gestational diabetes mellitus in Cameroon: prevalence, risk factors and screening strategies
Source: Front Clin Diabetes Healthc. 2024 Jan 9;4:1272333. doi: 10.3389/fcdhc.2023.1272333 (PMC10876121; doi:10.3389/fcdhc.2023.1272333)
Supplement: Supplementary file 3 [file Table_1.pdf]

Supplemental Table 1: Characteristics of the study population

**Characteristics**	**Values**
**N**	938
**Age, years**	25·5 ± 5·3
**Gestational age, weeks**	25·6 ± 1·7
**Education, %**	
Primary	55·8
Secondary	40·0
University	4·1
**Marital status, %**	
Single	19·7
Married/ In couple	79·8
Widow(er)/Separated/Divorced	0·4
**Tobacco smoking, %**	
Non-smokers	98·4
Ex-smokers	1·6
Current smokers	0·0
**Alcohol consumption, %**	
Non drinker	45·7
Ex-drinkers	28·4
Current drinkers	25·9
**Walking per week, %**	
< 30 min	54·3
30 – 60 min	29·1
> 60 min	16·6
**History of macrosomia, %**	9·9
**Known family history of diabetes, %**	15·5
**Number of previous live births, %**	
0	42·5
1 – 2	43·3
3 +	14·2
**Previous stillbirths, %**	2·6
**Current body mass index, kg/m^2**	27·6 ± 3·8
**Body mass index categories, %**	
Normal weight	25·0

Overweight	52·2
Obese	22·4
**Hip circumference, cm**	100·4 ± 10·6
**Systolic blood pressure, mmHg**	110·0 ± 10·6
**Diastolic blood pressure, mmHg**	63·3 ± 8·3
**High blood pressure, %**	1·5
**Fasting plasma glucose, mmol/L**	4·8 ± 0·8
**Two-hour post-glucose load glycaemia, mmol/L**	6·0 ± 1·1
